# Relationship between posaconazole concentrations and clinical outcomes in paediatric cancer and haematopoietic stem cell transplant recipients

**DOI:** 10.1093/jac/dkae473

**Published:** 2025-03-03

**Authors:** Heather Weerdenburg, Hannah Walker, Gabrielle M Haeusler, Theresa Cole, Nigel Curtis, Stephen Duffull, Amanda Gwee

**Affiliations:** Department of Pharmacy, Children’s Cancer Centre, General Medicine and Allergy and Immunology, Royal Children’s Hospital, Parkville, Australia; Antimicrobials, Clinical Paediatrics, and Infectious Diseases Groups, Murdoch Children’s Research Institute, Parkville, Australia; Department of Paediatrics, The University of Melbourne, Parkville, Australia; Department of Pharmacy, Children’s Cancer Centre, General Medicine and Allergy and Immunology, Royal Children’s Hospital, Parkville, Australia; Antimicrobials, Clinical Paediatrics, and Infectious Diseases Groups, Murdoch Children’s Research Institute, Parkville, Australia; Department of Paediatrics, The University of Melbourne, Parkville, Australia; Department of Pharmacy, Children’s Cancer Centre, General Medicine and Allergy and Immunology, Royal Children’s Hospital, Parkville, Australia; Antimicrobials, Clinical Paediatrics, and Infectious Diseases Groups, Murdoch Children’s Research Institute, Parkville, Australia; Department of Paediatrics, The University of Melbourne, Parkville, Australia; Department of Infectious Diseases, Peter MacCallum Cancer Centre, Parkville, Australia; Sir Peter MacCallum Department of Oncology, NHMRC National Centre for Infections in Cancer, University of Melbourne, Melbourne, Australia; The Victorian Paediatric Integrated Cancer Service, Victoria State Government, Melbourne, Australia; Department of Pharmacy, Children’s Cancer Centre, General Medicine and Allergy and Immunology, Royal Children’s Hospital, Parkville, Australia; Department of Paediatrics, The University of Melbourne, Parkville, Australia; Department of Pharmacy, Children’s Cancer Centre, General Medicine and Allergy and Immunology, Royal Children’s Hospital, Parkville, Australia; Antimicrobials, Clinical Paediatrics, and Infectious Diseases Groups, Murdoch Children’s Research Institute, Parkville, Australia; Department of Paediatrics, The University of Melbourne, Parkville, Australia; Certara, Princeton, NJ, USA; Department of Pharmacy, Children’s Cancer Centre, General Medicine and Allergy and Immunology, Royal Children’s Hospital, Parkville, Australia; Antimicrobials, Clinical Paediatrics, and Infectious Diseases Groups, Murdoch Children’s Research Institute, Parkville, Australia; Department of Paediatrics, The University of Melbourne, Parkville, Australia

## Abstract

**Background:**

Posaconazole is used to prevent and treat invasive fungal infections (IFIs) in immunocompromised children, including those undergoing cancer treatment or HSCT. Despite differences in pharmacokinetics and IFI epidemiology between children and adults, therapeutic targets established in adult studies are often applied to children.

**Objectives:**

This systematic review evaluated the correlation between serum posaconazole concentrations and clinical outcomes of IFI prophylaxis and treatment in children with malignancies or HSCT recipients.

**Methods:**

Four databases (Cochrane, Embase, MEDLINE and PubMed) were searched for studies involving children (≤18 years old) receiving cancer treatment or HSCT that reported posaconazole serum concentrations and treatment outcomes. Animal studies, those primarily in adult (>18 years old) populations, non-malignant conditions (excluding HSCT), case reports, letters, editorials, conference abstracts and narrative reviews were excluded. Bias was assessed using the Newcastle–Ottawa scale.

**Results:**

Nineteen studies were included: 12 reported outcomes of posaconazole prophylaxis; two of treatment; and five of both. For prophylaxis, breakthrough IFIs occurred in 1%–12% of children. All but one occurred with serum concentrations of ≤0.7 mg/L. For treatment, no clear association was observed between a trough concentration of >1.0 mg/L and treatment efficacy, with poor outcomes reported for serum concentrations ranging between 0.2 and 4.8 mg/L. Overall, quality of evidence was poor (medium to high risk of bias for 18 papers, low risk for 1 paper) and there was variation in IFI definitions across studies.

**Conclusions:**

This review supports current recommendations for posaconazole prophylaxis in paediatric oncology and HSCT recipients. The absence of a clear correlation found between serum trough concentrations and treatment efficacy highlights the need for further studies to determine optimal therapeutic targets for treatment.

## Introduction

Posaconazole, a mould-active antifungal agent, is used for the prevention and treatment of invasive fungal infections (IFIs) in children undergoing cancer treatment or HSCT.^1–4^ Current therapeutic guidelines suggest maintaining a serum trough concentration of at least 0.7 mg/L for prevention of IFI and 1.0–1.25 mg/L for treatment.^5^ These recommendations are based primarily on studies in adults who had a low incidence of treatment failure and breakthrough IFI.^5–8^

While exposure–response relationships are generally considered consistent across age groups, extrapolating evidence from adult studies to children is problematic for several reasons including notable differences in underlying diagnoses, chemotherapy regimens, comorbidities and pharmacokinetics.^9^ Children undergoing cancer treatment or HSCT have been shown to have a higher incidence and frequency of IFIs.^10–14^ Age also influences the infectious complications after HSCT, contributing to variations in IFI epidemiology and outcomes.^13^ Finally, susceptibility patterns of *Candida* spp. have been shown to differ between children and adults in some regions, highlighting unique challenges in managing IFIs in children and the need for tailored therapeutic approaches.^11,12,15^

The evidence for posaconazole efficacy and its exposure–response relationship is limited and higher posaconazole concentrations are assumed to be associated with improved outcomes.^5,8,16,17^ The aim of this systematic review was to evaluate the association between posaconazole concentrations and clinical outcomes for prophylaxis and treatment of IFI in children undergoing cancer treatment or HSCT.

## Methods

In December 2023, four databases (Cochrane, Embase, Medline and PubMed) were searched (see Table S1, available as Supplementary data at *JAC* Online, for search strategy) to identify articles reporting studies of posaconazole prophylaxis or treatment in children aged ≤18 years receiving cancer treatment or HSCT recipients that reported posaconazole serum concentrations and treatment outcomes. Animal studies, those primarily in adult (>18 years old) populations with paediatric data not reported separately, those in non-malignant conditions (excluding HSCT recipients), case reports, letters, editorials, conference abstracts and narrative reviews were also excluded. Titles and abstracts were independently reviewed by two investigators (H.We., H.Wa.) with discrepancies resolved in discussion with a third author (A.G.). Data on patient demographics, criteria for defining IFI (based on European Organisation for Research and Treatment of Cancer/Mycoses Study Group definitions^18^), fungal pathogen and serum posaconazole concentrations were extracted by a single investigator. Toxicity data were excluded from this review as this topic has been comprehensively addressed in a prior systematic review.^19^ Risk of bias was assessed by one reviewer using the Newcastle–Ottawa scale (NOS) for observational studies.^20^ Strength of studies was graded according to the five-point scale of the Oxford Centre for Evidence-Based Medicine (CEBM).^21^

## Results

**Figure 1.**
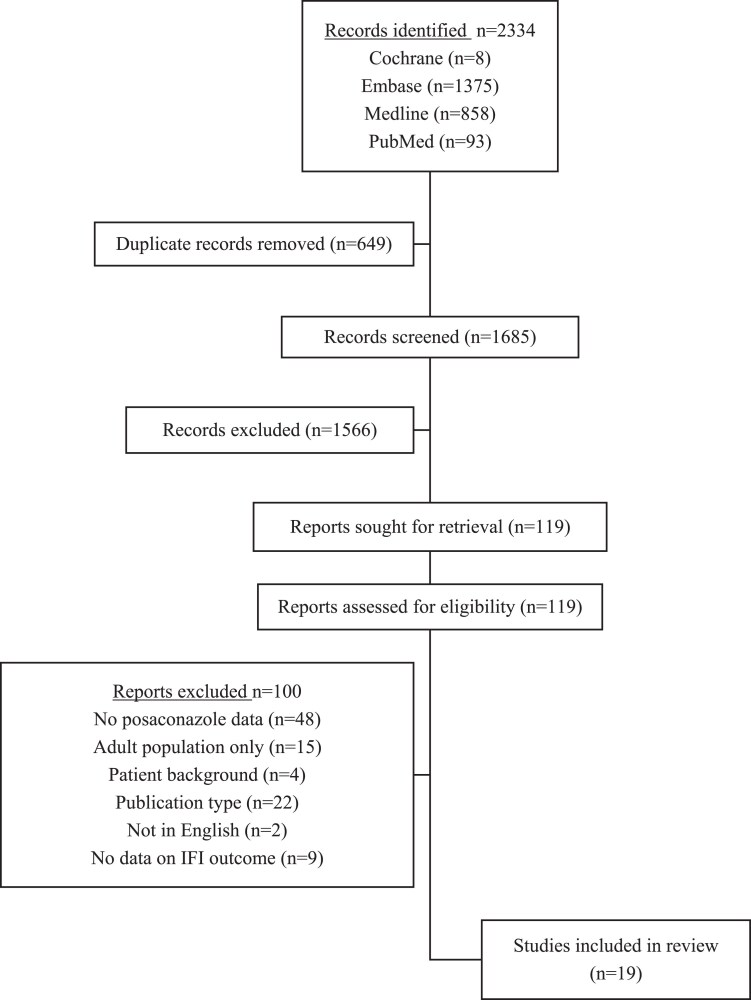
PRISMA flowchart for systematic literature search and study selection.

The search identified 2334 publications (Cochrane 8, Embase 1375, Medline 858, PubMed 93). After removal of 649 duplicates, 1685 abstracts and 119 full-text articles were reviewed for eligibility (Figure 1). Overall, 19 studies were included, of which 17 were retrospective and 2 were prospective. These studies detailed outcomes of prophylaxis (12 studies), treatment (2 studies) or both prophylaxis and treatment (5 studies) of IFIs.

Two methods of posaconazole monitoring were used in the included studies. Two studies reported the average steady-state concentration (*C*_avg_), with only one describing the target range of 0.5–2.5 mg/L.^2,22^ Seventeen studies measured the serum trough concentration (*C*_trough_) targeting ≥0.5 mg/L (1 study) and ≥0.7 mg/L (16 studies) for prophylaxis. For treatment, the *C*_trough_ target varied between studies: ≥0.7 mg/L (1 study);^23^ ≥1.0 (4 studies);^24–27^ and ≥1.2 mg/L (1 study).^28^

Of the seven studies reporting posaconazole use for salvage therapy, only four stated the fungal pathogen identified.^2,23,26,27^ Bias assessment using the NOS tool for included studies is detailed in Table S2. One study^29^ had a low risk of bias; the remaining 18 studies had a medium to high risk of bias.^23–40^

### Posaconazole prophylaxis

Of the 17 studies of posaconazole for IFI prophylaxis (Table 1), seven reported breakthrough infections occurring in 1%–12% of children, of whom all but two children had a posaconazole *C*_trough_ of <0.7 mg/L (Table 2).^25,26,29,31,33,35,38^ A retrospective audit by Jia *et al.*^25^ of 75 children with malignancy or HSCT recipients found 9 of the 75 children (12%) developed a proven or probable IFI despite 74% (203/273) of samples within the cohort showing a *C*_trough_ of ≥0.7 mg/L. Those with breakthrough IFI had significantly lower posaconazole concentrations in comparison with those without an IFI [median 0.43 (range 0.1–0.7) versus 1.2 mg/L (range 0.1–2.1), respectively; *P* < 0.001]^25^ This correlation was also observed in another retrospective review by Lai *et al.*^35^ of 70 children that reported lower mean *C*_trough_ levels in the three (4%) children with breakthrough probable IFI, compared with those without infection (0.6 versus 0.9 mg/L, respectively; *P* = 0.002). The three children who developed breakthrough IFI had *C*_trough_ concentrations ranging between 0.4 and 0.6 mg/L. Unfortunately, neither study reported the IFI diagnostic criteria or IFI risk factors.

**Table 1.** Posaconazole prophylaxis

Paper ; study design	Population	Posaconazole target achieved	Posaconazole: formulation, dosing	Median course (range)	Key findings	Risk of bias	Grade of evidence^a^
Bernardo *et al.*^24^ Retrospective single-centre study	23 children 2–21 years^b^ Malignancy & HSCT	≥0.7 mg/L 65% (*n* = 15/23)	DRT & IV 10 mg/kg q24h (max 300 mg)	51 days (8–306)	No breakthrough IFI No death	Medium/high	3
Döring *et al.*^30^ Retrospective single-centre study	33 children 1–17 years Malignancy, BMF or ID	≥0.7 mg/L 12% (*n* = 20/172 samples)	Suspension 4 mg/kg q8h	79 days (24–214)	IFI breakthrough: 3% (1/33) *A. fumigatus* (*C*_trough_: 0.3 mg/L) Outcome: complete response No death	Medium/high	3
Döring *et al.*^31^ Retrospective single-centre study	63 children 6 months–18 years HSCT	≥0.5 mg/L Suspension 29% (*n* = 9/31) DRT 77% (*n* = 31/32)	Suspension 4 mg/kg q8h DRT 100–300 mg q24h	Suspension 108 days (41–206) DRT 106 (44–203)	No breakthrough IFI No death	Medium/high	3
Döring *et al.*^28^ Retrospective single-centre study	60 children 8 months–11 years HSCT	NR	Suspension 5 mg/kg q12h (28) 4 mg/kg q8h (32)	127 days (12–188)	No breakthrough IFI Death NR	Medium/high	3
Duehlmeyer *et al.*^37^ Single site retrospective study	5 children 7–19 years^b^ Malignancy or ID	≥0.7 mg/L 80% (*n* = 4/5)	Suspension: <12 years: 4 mg/kg q8h DRT: ≥12 years: 200 mg or 300 mg q24h	NR	No breakthrough IFI No death	Medium/high	3
Garner *et al.*^36^ Single-centre retrospective study	13 children 1–18 years Malignancy, ID, HSCT or other	≥0.7 mg/L 77% (*n* = 10/13)	Suspension 10 mg/kg/day DRT 200–300 mg/day Injection: 10–12 mg/kg/day	NR	No breakthrough IFI Death NR	Medium/high	3
Heinz *et al.*^40^ Single-centre retrospective study	27 children 1–16 years Malignancy or ID	>0.7 mg/L 65% (*n* = 103/161 samples)	Suspension 4 mg/kg q8h	Prophylaxis: 112 days (22–200)	No breakthrough IFI Death 11% (*n* = 3/27^c^)	Medium/high	3
Jia *et al.*^25^ Single-centre retrospective study	75 children 1–18 years (median 10 years) Malignancy, BMF, ID & HSCT	≥0.7 mg/L 74% (*n* = 203/273 samples)	Suspension Dose NR	84 days (7–320)	IFI breakthrough 12% (*n* = 9/75)^d^ Death NR	Medium/high	3
Lai *et al.*^35^ Single-centre retrospective study	70 children 3 months–12 years (median 5 years) Malignancy, BMF, ID, HSCT	≥0.7 mg/L 48% (*n* = NR)	Prophylaxis: Suspension 5 mg/kg (max 200 mg) q8h	Prophylaxis: 88 days (32–168)	IFI breakthrough 4% (3/70) Probable: *n* = 3/3 *C*_trough_ range 0.4 and 0.6 mg/L) Death NR	Medium/high	3
Mathew *et al.*^33^ Single-centre retrospective study	32 children 3–18 years Malignancy, BMF, ID	≥0.7 mg/L 50% (*n* = 16/32)	Prophylaxis: Suspension <13 years: 10–12 mg/kg/day ≥13 years: 600–800 mg/day	NR	IFI breakthrough 7% (*n* = 1/15) Proven cutaneous mould *C*_trough_ of 0.3 mg/L Death 3% (*n* = 1/32)	Medium/high	3
Mauro *et al.*^27^ Multicentre retrospective study	21 children 5–18 years median 15 years Malignancy, BMF, HSCT	≥0.7 mg/L 85% (*n* = 22/26 samples)	DRT <50 kg: 4–6 mg/kg q24h ≥50 kg: 300 mg q24h	Suspension 108 days (41–206) DRT 106 days (45–203)	No breakthrough IFI No death	Medium/high	3
McMahon *et al.*^34^ Single-centre retrospective study	13 children 1–12 years HSCT	>0.7 mg/L 50% (*n* = 5/10)	Suspension <30kg: 4 mg/kg q8h ≥30 kg: 200 mg q8h	10 days (6–29)	No breakthrough IFI Death NR	Medium/high	3
Takpradit *et al.*^29^ Single site retrospective study	41 children 1–17 years HSCT	≥0.7 mg/L 19% (*n* = 7/37)	Suspension 4 mg/kg q8h	NR	IFI breakthrough 5% (*n* = 2/41) Probable *n* = 2/41 (*C*_trough_ 0.6 and 0.5 mg/L) No death	Low	3
Tragiannidis *et al.*^32^ Retrospective single-centre study	34 children 5–17 years BMF, HSCT	≥0.7 mg/L 94% (*n* = 32/34)	DRT 200–300 mg q24h	70 days (9–391)	No breakthrough IFI Death NR	Medium/high	3
Vanstraelen *et al.*^39^ Prospective single site study	14 children 2–13 years Malignancy, HSCT	≥0.7 mg/L 57% (*n* = 8/14)	Suspension 4.6 mg/kg q8h	21 days (17–60)	No breakthrough IFI Death NR	Medium/high	3
Vicenzi *et al.*^26^ Multicentre retrospective study	79 children 4 months–18 years Malignancy or HSCT	≥0.7 mg/L 63% (*n* = 50/79)	Suspension Median 10–12 mg/kg/day (4–40)	148 days (11–1117)	5% (*n* = 4/84) Probable: *n* = 1/4 Possible: *n* = 3/4 *C*_trough_ range 0.1–0.6 mg/L (*n* = 3/4)^e^ No deaths	Medium/high	3
Wass *et al.*^38^ Retrospective single site study	65 children NR (up to 17 years) Malignancy or HSCT	≥0.7 mg/L 56% (*n* = 197/353 samples)	Suspension & DRT Dose NR	NR	IFI breakthrough 1.5% (*n* = 1/65) *R. mucilaginosa* (*C*_trough_ 0.7 mg/L) Death: 1.5% (*n* = 1/65)	Medium/high	3

BMF, bone marrow failure; DRT, delayed-release tablet; ID, immunodeficiency; NR, Not reported.

^a^Using grading as per Oxford CEBM.

^b^Unable to exclude >18 years for study.

^c^Death reported due to underlying malignancy.

^d^No further details on breakthrough IFI.

^e^ *C*_trough_ for one child not documented; however, reported to be ≥0.7 mg/L.

**Table 2.** Studies with IFI breakthrough on posaconazole and reported *C*_trough_ achieved

Study	Breakthrough IFI (%)	*C*_trough_ <0.5 mg/L	*C*_trough_ 0.5 to <0.7 mg/L	*C_t_*_rough_ ≥0.7 mg/L	Fungal pathogen
Döring *et al.*^30^	1/33 (3)	1	0	0	*A. fumigatus* ^a^
Jia *et al.*^25^	9/75 (12)^b^	9/9^c^			Not reported
Lai *et al.*^35^	3/70 (4)^d^	1/3	2/3	0	Not reported
Mathew *et al.*^33^	1/15 (7)	1	0	0	Not reported
Takpradit *et al.*^29^	2/37 (5)	0	2	0	Not reported
Vicenzi *et al.*^26^	4/85 (5)	3/4		1/4^e^	Not reported
Wass *et al.*^38^	1/65 (2)	1	0	0	*R. mucilaginose* ^f^

^a^Primary site of IFI: appendix.

^b^Children with a breakthrough IFI: *C*_trough_ median 0.4 mg/L (range 0.1–0.7) versus no IFI breakthrough: 1.2 mg/L (range 0.1–2.1); *P* < 0.001.

^c^Individual *C*_trough_ not reported: *C*_trough_ range reported: 0.1–0.7 mg/L.

^d^Mean *C*_trough_ with IFI breakthrough: 0.6 mg/L versus no IFI breakthrough: 0.9 mg/L (*P* = 0.002).

^e^Individual *C*_trough_ reported as therapeutic (≥0.7 mg/L but not documented).

^f^Blood culture.

A retrospective study by Döring *et al.*^30^ of 33 children with malignancy and bone marrow failure reported one (3%) breakthrough infection despite only 12% (20/172) of samples reaching the target *C*_trough_ of ≥0.7 mg/L. This child with IFI had a history of bone marrow failure and cultured *Aspergillus fumigatus* from their appendix in the setting of having a *C*_trough_ of 0.3 mg/L. In a small study of 15 children by Mathew *et al.*,^33^ one patient (7%) developed a proven and fatal cutaneous mould infection with elevated 1,3-β-D-glucan level and a raised serum galactomannan level, with a low posaconazole *C*_trough_ of 0.3 mg/L. Similarly, another retrospective study in 37 HSCT recipients (Takpradit *et al.*^29^) reported only two (5%) probable IFIs despite low overall attainment of target concentrations (19% with *C*_trough_ of ≥0.7 mg/L). Both children had subtherapeutic serum concentrations (0.6 and 0.5 mg/L).

Two retrospective studies reported breakthrough IFI in children with a *C*_trough_ of ≥0.7 mg/L. Wass *et al.*^38^ observed one case of IFI occurring while on posaconazole prophylaxis in 65 children with malignancy or HSCT recipients. The child had ALL and developed a fatal *Rhodotorula mucilaginosa* infection with a *C*_trough_ of 0.7 mg/L. Notably, 27/65 of the included children received concomitant antifungal therapy with their posaconazole prophylaxis.^36^ In the second study by Vicenzi *et al.*,^26^ 4/84 (5%) children, of whom two had haematological malignancy and two were HSCT recipients, developed pulmonary IFI: one probable (positive serum galactomannan) and three possible (note one patient was positive for serum β-D-glucan but this was not included in their definition of probable). All four children were neutropenic at the time of IFI diagnosis and three had a posaconazole *C*_trough_ of <0.7 mg/L (range 0.1–0.6). Clinical details of the fourth child who had a therapeutic level wasn’t reported.

No instances of breakthrough IFI occurred in the remaining 10 studies. Of these, two studies reported low serum posaconazole concentrations: one (Döring *et al.*^31^) targeted a *C*_trough_ of ≥0.5 mg/L; and the other (Döring *et al.*^28^) reported a median posaconazole concentration of 0.1–0.4 mg/L. Eight studies reported achieving a higher attainment of posaconazole target concentrations (≥0.7 mg/L) in 50%–100% of children with no reported IFI breakthrough infections.^24,27,32,34,36,37,39,40^

### Posaconazole for treatment of IFI

Outcomes of IFI treatment with posaconazole were reported in seven small studies that included between 7 and 33 patients (Table 3).^2,23–27,37^ A multicentre trial by Krishna *et al.*^2^ of immunocompromised children requiring posaconazole treatment due to intolerance or resistance to other antifungals found 5/8 (63%) had a complete or partial response determined by a blinded expert committee. Fungal pathogens isolated in those who responded to treatment included *Aspergillus* spp., *Candida krusei*, *Coccidioides immitis* and *Scedosporium apiospermum.* The remaining three children who failed therapy had infections caused by *Candida* spp., *Aspergillus* spp. and *Fusarium* spp. There was no significant difference in the *C*_avg_ observed between those who responded and those that didn’t (0.1–1.0 mg/L versus 0.1–1.2 mg/L, respectively). *In vitro* susceptibility to posaconazole was not documented.

**Table 3.** Posaconazole treatment

Paper ; study design	Population	Posaconazole target, % achieved	Posaconazole formulation & dosing	Median course (range)	Key findings	Risk of bias^a^	Evidence grade^b^
Bernardo *et al.*^23^ Retrospective single-centre study	33 children 6 months–23 years^c^ Malignancy or ID IFI diagnosis: Empirical: *n* = 19 Proven/probable: *n* = 14	≥0.7 mg/L 64 (*n* = 21/33)	Suspension <34 kg: 4.5–6 mg/kg q6h ≥13 years &/or ≥34 kg: 200 mg q6h	NR	Overall response: *n* = 33 1. Empirical: (*n* = 19) Failure: 5% (*n* = 1/19, *C*_trough_: 0.9 μg/mL) 2. Proven/probable: (*n* = 14) Failure: 21% (*n* = 3/14) *Aspergillus* spp. (eyes & lungs) *C. albicans & C. famata* (hard palate, left maxilla, nose, and left orbit)^d^ *Zygomycete* spp. (pulmonary) Death: *n* = 2 Child 1 *C*_trough:_ 0.2 & 0.7 mg/L. Child 2: 0.6 & 1.6 mg/L	Medium/high	3
Bernardo *et al.*^24^ Retrospective single-centre study	31 children 2–21 years^c^ Malignancy & HSCT IFI diagnosis criteria: EORTC-MSG Empirical: *n* = 4 Possible: *n* = 19 Proven: *n* = 8	Treatment: ≥1.0 mg/L 68 (*n* = 21/31)	DRT & IV ≥13 years: 300 mg q24h <13 years: 10 mg/kg q24h	51 days (8–306)	Overall response 1. Empirical Resolved: 100% (*n* = 4) 2. Possible^e^ Complete/partial/stable:79% (*n* = 17/19) IFI ruled out: 11% 2/19 3. Proven IFI Complete/partial: 63% (5/8) Death: *n* = 3^f^	Medium/high	3
Duehlmeyer *et al.*^37^ Single-site retrospective study	11 children 7–19 years^c^ Malignancy or ID IFI diagnosis criteria not specified	Treatment: ≥1.2 mg/L 91 (*n* = 10/11)	Suspension: <12 y: 4 mg/kg q8h DRT: ≥ 12 years: 200 mg or 300 mg q24h	NR	Overall response Positive (*n* = 9/11) median *C*_trough_: 1.2 mg/L (0.1–5.0) Failure: 18% (*n* = 2/11)^c^ median *C*_trough_: 0.5 mg/L (0.2–2.5) Death: 18% (*n* = 2/11)^c^	Medium/high	3
Krishna *et al.*^2^ Multicentre, Phase 3, open-label study	8 children^g^ 8–17 years Malignancy, HSCT, or other ID IFI diagnosis criteria: EORTC-MSG) Proven/probable: *n* = 8	*C*_avg_ No target	Suspension 200 mg q6h or 400 mg q12h	NR	Overall response Complete/partial: 63% (*n* = 5/8) Failure: 37% (*n* = 3/8) Pathogens: *Aspergillus* spp. (*n* = 3) *Fusarium* spp. (*n* = 1) *Candida* spp. (*n* = 2) *Lomentospora + Aspergillus* spp. (*n* = 1) *C. immitis* (*n* = 1) Death: *n* = 1^h^	Medium/high	3
Jia *et al.*^25^ Single-centre retrospective study	24 children 1–18 years (median 10 years) Malignancy, BMF, ID or HSCT IFI diagnosis criteria: EORTC-MSG but not individually reported	≥1.0 mg/L 48 (*n* = 51/102 samples)	Suspension Dose NR	84 days (7–320)	IFI criteria: EORTC-MSG Overall response Complete/partial: 79% (*n* = 21/24)^i^ Median *C*_trough_: 1.1 mg/L Failure: 21% (*n* = 5/24) Median *C*_trough_: 0.5 mg/L Death: nil	Medium/high	3
Mauro *et al.*^27^ Multicentre retrospective study	7 children 5–18 years median 15 years Malignancy, BMF, HSCT IFI diagnosis criteria not specified	≥1.0 mg/L 63 (*n* = 9/14)	DRT <50 kg: 4–6 mg/kg q24h ≥50 kg: 300 mg q24h	21 days (range 7–204)	Overall response Complete/partial: 58% (*n* = 4/7) Stable:14% (*n* = 1/7) Failure: 14% (*n* = 1/7) Not recorded: 14% (*n* = 1/7) Proven pathogen identified (*n* = 3) *Aspergillus* (*n* = 2) *A. corymbifera* (*n* = 1) Death: nil	Medium/high	3
Vicenzi *et al.*^26^ Multicentre retrospective study	13 children 4 months–18 years Malignancy or HSCT IFI diagnosis criteria: EORTC-MSG Proven/probable: *n* = 5 Possible: *n* = 8	≥1.0 mg/L 71 (*n* = 20/28 samples)	Suspension Median 10–12 mg/kg/day (4–40)	Median 147 days (20–687)	Overall response Complete/partial response:77% (*n* = 10/13) Stable response: 8% (*n* = 1/13) Failure: 8% (*n* = 1/13) Proven pathogens identified (*n* = 9) *Aspergillus* spp. (*n* = 5/9) *Mucorales* spp. (*n* = 2/9) Unknown filamentous hyphae (*n* = 2/9) Death (*n* = 1)	Medium/high	3

BMF, bone marrow failure; *C*_avg_, AUC/dosing interval; DRT, delayed-release tablet; EORTC-MSG, European Organization for Research and Treatment of Cancer/Mycoses Study Group;^18^ ID, immunodeficiency; NR, not reported.

^a^Risk of bias: the maximum overall score is nine stars for studies where comparability was assessed and seven stars for studies where comparability was not assessed (a maximum of one star can be earned for each item in the scale with the exception of comparability where a maximum of two stars can be earned). Studies with a score of ≥7 stars were considered to have a low risk of bias, and those with scores of ≤7 had a medium or high risk of bias.

^b^Using grading as per Oxford CEBM.

^c^Unable to exclude >18 years for study.

^d^Patient on concomitant antifungal treatment with micafungin and liposomal amphotericin.

^e^Possible IFI and started on therapy after a CT scan showed concerns for fungal infection.

^f^Cause of death: IFI (*n* = 2) with posaconazole length of treatment 7 days and 13 days. Underlying disease (*n* = 1).

^g^Only children included who completed the study

^h^Death reported due to underlying malignancy.

^i^Further details on positive response not reported.

The remaining six studies monitored posaconazole trough concentrations: five targeted a *C*_trough_ of ≥1.0–1.25 mg/L and one a *C*_trough_ of ≥0.7 mg/L. A retrospective study by Jia *et al.*^25^ reported successful outcomes in 19/24 children (79%). Treatment failure in five children was associated with a lower median *C*_trough_ compared with those who responded to treatment: 0.5 (range 0.3–0.7) versus 1.1 mg/L (range 0.6–3.1), respectively (*P* < 0.024). There were no IFI-related deaths reported. Similarly, a study by Duehlmeyer *et al.*^37^ of 11 children receiving posaconazole therapy for IFI reported that two had progression leading to death. The median posaconazole concentration was lower in those who died (0.5 mg/L, range 0.2–2.5) compared with survivors (1.2 mg/L, range 0.1–5.0). Individual trough concentrations were not reported.

In contrast, a study of 33 children with possible or probable (19) and proven (14) IFI (Bernardo *et al.*^23^) targeted a lower *C*_trough_ of ≥0.7 mg/L. This study found that IFI progression in four children was not associated with the posaconazole plasma concentrations (*P* > 0.05); C_trough_ values were 0.2–1.6 mg/L. Two IFI-related deaths occurred: one with disseminated *Aspergillus* spp. infection who had low *C*_trough_ (0.2 and 0.7 mg/L); the other with disseminated *Candida albicans* and *Candida famata* who received concomitant liposomal amphotericin and micafungin and whose *C*_trough_ ranged between 0.6 and 1.6 mg/L. Posaconazole concentrations were reported in 14 (42%) children in this study.

The remaining three studies found no clear correlation between posaconazole concentrations and clinical outcome. A retrospective study (Bernardo *et al.*^24^) included 31 children treated with posaconazole for empirical (4), suspected (19) or proven (8) IFI. The median posaconazole plasma concentration was 1.5 mg/L (range 0.5–7.1). Among the children, 26 showed complete, partial or stable responses, and IFI was ruled out in two cases. The remaining three died: one due to progressive IFI while on posaconazole (7 days); another after switching from posaconazole (13 days) to voriconazole and micafungin; and the third due to an underlying disease. Posaconazole concentrations were not individually reported for these children. Similarly, Mauro *et al.*^27^ described seven children with IFI (three with proven pathogens; two *Aspergillus* spp. and one *Absidia corymbifera*). The majority were pulmonary infections, with one involving the facial skeleton. Four had a complete or partial response, one had stable disease, and one had progression with posaconazole *C*_trough_ values between 0.7 and 4.8 mg/L (outcomes for one patient not reported). In a retrospective study by Vicenzi *et al.*^26^ of 13 children receiving salvage posaconazole therapy for proven or probable (5) or possible (8) IFI, 71% (20/28) of samples taken achieved a *C*_trough_ of ≥1.0 mg/L. Most (11) children received dual antifungal therapy. Treatment outcomes varied, with a complete response in two, partial response in eight, and stable disease in one child. There was one treatment failure and one death due to underlying disease. Individual *C*_trough_ levels were not reported.

## Discussion

To our knowledge, this is the first systematic review evaluating the clinical outcomes observed with the current recommended targets for therapeutic drug monitoring of posaconazole in paediatric oncology or HSCT recipients. Our findings support existing guidelines for posaconazole prophylaxis, showing that breakthrough fungal infections primarily occurred when trough concentrations were 0.7 mg/L or below (evidence level 3, medium to high risk of bias). In contrast, studies using posaconazole for treatment did not consistently demonstrate a clear association between achieving trough concentrations of ≥1.0 mg/L and treatment outcomes. The current therapeutic targets for posaconazole prophylaxis originated from an exposure–response analysis of data from two clinical trials in adults comparing posaconazole prophylaxis with fluconazole and itraconazole in adult HSCT and cancer patients. This found a clear association between posaconazole and clinical failure: average concentrations of 0.3, 0.7, 1.2 and 2.6 mg/L were associated with clinical failure at a frequency of 44%, 21%, 18% and 18%, respectively.^41–43^ Further cohort studies in adults have also supported the posaconazole prophylaxis *C*_trough_ target of ≥0.7 mg/L.^44–48^

A lower prophylaxis trough target of 0.5 mg/L has been suggested in several papers citing posaconazole’s unique distribution pattern with 40-fold higher concentrations in pulmonary alveolar cells than in plasma. However, there is limited direct evidence linking intracellular concentrations to prophylactic efficacy.^49,50^ International guidelines have continued to recommend higher targets due to posaconazole’s favourable safety profile and the potential for extrapulmonary IFI.^8,50,51^ This review highlights the importance of maintaining a *C*_trough_ above 0.7 mg/L in children, given the high occurrence of breakthrough IFIs below this concentration cut-off.^25,26,29–31,33,35,38^ In contrast to prophylaxis, the optimal therapeutic target for IFI treatment with posaconazole is less clear. Current therapeutic treatment targets (*C*_trough_ ≥ 1.0–1.25 mg/L) are based on adult studies showing a correlation between posaconazole exposure and treatment response in refractory IFI.^7^ In patients with invasive *Aspergillus* spp., a higher posaconazole plasma concentration correlated with improved clinical response rates.^7^ However, consistent with our review, two studies in adults reporting the use of posaconazole for treatment of chronic disseminated coccidioidomycosis and pulmonary aspergillosis found a poor correlation between trough concentrations and treatment outcomes.^52,53^ This underscores the complexity of treating IFIs as several factors beyond drug concentrations significantly impact treatment outcomes. These include the extent of disease, concomitant treatments including surgical debridement, and the underlying immunosuppressive state.^10,44^ Preclinical animal models that closely replicate human disease and control for these variables could help clarify exposure–response relationships and inform optimal therapeutic targets.

Limitations of our review include the retrospective nature of the majority (17/19) of studies, small sample size and the moderate to high level of bias identified in almost all studies (18/19). Additionally, factors that may also influence the clinical outcome of IFI, including duration and/or severity of neutropenia, use of immunosuppression (e.g. corticosteroids) and complementary therapy such as surgical excision, were not reported in several studies. The incomplete data on posaconazole levels could have contributed to the lack of observed association between plasma concentrations and IFI progression. While MIC data are not always essential due to the absence of a clear MIC–outcome relationship for many fungal organisms, the lack of consistent criteria for diagnosing IFIs and defining treatment outcomes remains a significant barrier to interpreting the results.

In conclusion, the findings of this systematic review reinforce current recommendations for posaconazole prophylaxis, with evidence supporting posaconazole concentrations of >0.7 mg/L in paediatric oncology and HSCT recipients. There is a lack of evidence supporting an association between posaconazole trough concentrations and efficacy in treating IFIs. Future efforts should focus on more targeted approaches, such as utilizing preclinical pharmacokinetic/pharmacodynamic (PK/PD) assessments and exploring patient-specific factors to provide valuable insights to help optimize treatment outcomes.

## Supplementary Material

dkae473_Supplementary_Data

## Supplementary data

Tables S1 and S2 are available as Supplementary data at *JAC* Online.
