# Supplementary material for: Relationship between posaconazole concentrations and clinical outcomes in paediatric cancer and haematopoietic stem cell transplant recipients
Source: J Antimicrob Chemother. 2025 Mar 3;80(4):897–907. doi: 10.1093/jac/dkae473 (PMC11962376; doi:10.1093/jac/dkae473)
Supplement: dkae473_Supplementary_Data [file dkae473_supplementary_data.docx]

Table S1: Posaconazole search strategy

| **Embase: 1947 - 2023** | |
| --- | --- |
| 1. | posaconazole/ |
| 2. | (Posaconazole or noxafil or sch-56592 or sch56592 or spriafil).tw,kf,dq. |
| 3. | (newborn* or new-born* or baby or babies or neonat* or neo-nat* or infan* or toddler* or pre-schooler* or preschooler* or kinder or kinders or kindergarten* or kinder-aged or boy or boys or girl or girls or child or children or childhood or pediatric* or paediatric* or school-age* or schoolage* or schoolchild* or schoolgirl* or schoolboy* or adolescent* or youth or youths or teen or teens or teenage*).af |
| 4. | (rat or rats or mouse or mice or rodent* or swine or porcine or murine or sheep or lamb or lambs or pig or pigs or piglet or piglets or rabbit or rabbits or cat or cats or dog or dogs or cattle or bovine or monkey or monkeys or trout or marmoset or marmosets).ti. and animal experiment/ |
| 5. | Animal experiment/ not (human experiment/ or human/) |
| 6. | (1 or 2) and 3 |
| 7. | 6 not (4 or 5) |
| 8. | Case report |
| 9. | limit 7 to (conference abstract or conference paper or "conference review" or editorial or letter) |
| 10. | 7 not (8 or 9) |
| **Medline: 1947 - 2023** | |
| Set | Search statement |
| 1. | (Posaconazole or noxafil or sch-56592 or sch56592 or spriafil).tw,kf. |
| 2. | (newborn* or new-born* or baby or babies or neonat* or neo-nat* or infan* or toddler* or pre-schooler* or preschooler* or kinder or kinders or kindergarten* or kinder-aged or boy or boys or girl or girls or child or children or childhood or pediatric* or paediatric* or school-age* or schoolage* or schoolchild* or schoolgirl* or schoolboy* or adolescen* or youth or youths or teen or teens or teenage*).af. |
| 3. | (exp animals/ or (rat or rats or mouse or mice or rodent* or swine or porcine or murine or sheep or lamb or lambs or pig or pigs or piglet or piglets or rabbit or rabbits or cat or cats or dog or dogs or cattle or bovine or monkey or monkeys or trout or marmoset or marmosets).ti.) not human*.sh. |
| 4. | (1 and 2) not 3 |
| 5. | limit 4 to (case reports or comment or editorial or letter) |
| 6. | 4 not 5 |
| **PubMed: 2006 - 2023** | |
| Set | Search statement |
| 1. | (((("Posaconazole"[Title/Abstract] OR "noxafil"[Title/Abstract] OR "sch-56592"[Title/Abstract] OR "sch56592"[Title/Abstract] OR "spriafil"[Title/Abstract]) AND ("newborn*"[Title/Abstract] OR "new-born*"[Title/Abstract] OR "baby"[Title/Abstract] OR "babies"[Title/Abstract] OR "neonat*"[Title/Abstract] OR "neo-nat*"[Title/Abstract] OR "infan*"[Title/Abstract] OR "toddler*"[Title/Abstract] OR "pre-schooler*"[Title/Abstract] OR "preschooler*"[Title/Abstract] OR "kinder"[Title/Abstract] OR "kinders"[Title/Abstract] OR "kindergarten*"[Title/Abstract] OR "kinder-aged"[Title/Abstract] OR "boy"[Title/Abstract] OR "boys"[Title/Abstract] OR "girl"[Title/Abstract] OR "girls"[Title/Abstract] OR "child"[Title/Abstract] OR "children"[Title/Abstract] OR "childhood"[Title/Abstract] OR "pediatric*"[Title/Abstract] OR "paediatric*"[Title/Abstract] OR "school-age*"[Title/Abstract] OR "schoolage*"[Title/Abstract] OR "schoolchild*"[Title/Abstract] OR "schoolgirl*"[Title/Abstract] OR "schoolboy*"[Title/Abstract] OR "adolescen*"[Title/Abstract] OR "youth"[Title/Abstract] OR "youths"[Title/Abstract] OR "teen"[Title/Abstract] OR "teens"[Title/Abstract] OR "teenage*"[Title/Abstract]) AND (NOTNLM OR publisher[sb] OR inprocess[sb] OR pubmednotmedline[sb] OR indatareview[sb] OR pubstatusaheadofprint)) NOT (("Animal" OR "animals" OR "rat" OR "rats" OR "mouse" OR "mice" OR "rodent*" OR "swine" OR "porcine" OR "murine" OR "sheep" OR "lamb" OR "lambs" OR "pig" OR "pigs" OR "piglet" OR "piglets" OR "rabbit" OR "rabbits" OR "cat" OR "cats" OR "dog" OR "dogs" OR "cattle" OR "bovine" OR "monkey" OR "monkeys" OR "trout" OR "marmoset" OR "marmosets") NOT ("human" OR "humans" OR "patient" OR "patients" OR "newborn*" OR "baby" OR "babies" OR "neonat*" OR "infan*" OR "toddler*" OR "pre-schooler*" OR "preschooler*" OR "kindergarten" OR "boy" OR "boys" OR "girl" OR "girls" OR "child" OR "children" OR "childhood" OR "adolescen*" OR "pediatric*" OR "paediatric*" OR "youth*" OR "teen" OR "teens" OR "teenage*" OR "school-aged*" OR "school-child*" OR "school-girl*" OR "school-boy*" OR "schoolgirl*" OR "schoolboy*" OR "man" OR "men" OR "woman" OR "women" OR "adult" OR "adults" OR "middle-age*" OR "elderly"))) NOT ((((("Posaconazole"[Title/Abstract] OR "noxafil"[Title/Abstract] OR "sch-56592"[Title/Abstract] OR "sch56592"[Title/Abstract] OR "spriafil"[Title/Abstract]) AND ("newborn*"[Title/Abstract] OR "new-born*"[Title/Abstract] OR "baby"[Title/Abstract] OR "babies"[Title/Abstract] OR "neonat*"[Title/Abstract] OR "neo-nat*"[Title/Abstract] OR "infan*"[Title/Abstract] OR "toddler*"[Title/Abstract] OR "pre-schooler*"[Title/Abstract] OR "preschooler*"[Title/Abstract] OR "kinder"[Title/Abstract] OR "kinders"[Title/Abstract] OR "kindergarten*"[Title/Abstract] OR "kinder-aged"[Title/Abstract] OR "boy"[Title/Abstract] OR "boys"[Title/Abstract] OR "girl"[Title/Abstract] OR "girls"[Title/Abstract] OR "child"[Title/Abstract] OR "children"[Title/Abstract] OR "childhood"[Title/Abstract] OR "pediatric*"[Title/Abstract] OR "paediatric*"[Title/Abstract] OR "school-age*"[Title/Abstract] OR "schoolage*"[Title/Abstract] OR "schoolchild*"[Title/Abstract] OR "schoolgirl*"[Title/Abstract] OR "schoolboy*"[Title/Abstract] OR "adolescen*"[Title/Abstract] OR "youth"[Title/Abstract] OR "youths"[Title/Abstract] OR "teen"[Title/Abstract] OR "teens"[Title/Abstract] OR "teenage*"[Title/Abstract]) AND (NOTNLM OR publisher[sb] OR inprocess[sb] OR pubmednotmedline[sb] OR indatareview[sb] OR pubstatusaheadofprint)) NOT (("Animal" OR "animals" OR "rat" OR "rats" OR "mouse" OR "mice" OR "rodent*" OR "swine" OR "porcine" OR "murine" OR "sheep" OR "lamb" OR "lambs" OR "pig" OR "pigs" OR "piglet" OR "piglets" OR "rabbit" OR "rabbits" OR "cat" OR "cats" OR "dog" OR "dogs" OR "cattle" OR "bovine" OR "monkey" OR "monkeys" OR "trout" OR "marmoset" OR "marmosets") NOT ("human" OR "humans" OR "patient" OR "patients" OR "newborn*" OR "baby" OR "babies" OR "neonat*" OR "infan*" OR "toddler*" OR "pre-schooler*" OR "preschooler*" OR "kindergarten" OR "boy" OR "boys" OR "girl" OR "girls" OR "child" OR "children" OR "childhood" OR "adolescen*" OR "pediatric*" OR "paediatric*" OR "youth*" OR "teen" OR "teens" OR "teenage*" OR "school-aged*" OR "school-child*" OR "school-girl*" OR "school-boy*" OR "schoolgirl*" OR "schoolboy*" OR "man" OR "men" OR "woman" OR "women" OR "adult" OR "adults" OR "middle-age*" OR "elderly"))) AND (booksdocs[Filter])) OR (((("Posaconazole"[Title/Abstract] OR "noxafil"[Title/Abstract] OR "sch-56592"[Title/Abstract] OR "sch56592"[Title/Abstract] OR "spriafil"[Title/Abstract]) AND ("newborn*"[Title/Abstract] OR "new-born*"[Title/Abstract] OR "baby"[Title/Abstract] OR "babies"[Title/Abstract] OR "neonat*"[Title/Abstract] OR "neo-nat*"[Title/Abstract] OR "infan*"[Title/Abstract] OR "toddler*"[Title/Abstract] OR "pre-schooler*"[Title/Abstract] OR "preschooler*"[Title/Abstract] OR "kinder"[Title/Abstract] OR "kinders"[Title/Abstract] OR "kindergarten*"[Title/Abstract] OR "kinder-aged"[Title/Abstract] OR "boy"[Title/Abstract] OR "boys"[Title/Abstract] OR "girl"[Title/Abstract] OR "girls"[Title/Abstract] OR "child"[Title/Abstract] OR "children"[Title/Abstract] OR "childhood"[Title/Abstract] OR "pediatric*"[Title/Abstract] OR "paediatric*"[Title/Abstract] OR "school-age*"[Title/Abstract] OR "schoolage*"[Title/Abstract] OR "schoolchild*"[Title/Abstract] OR "schoolgirl*"[Title/Abstract] OR "schoolboy*"[Title/Abstract] OR "adolescen*"[Title/Abstract] OR "youth"[Title/Abstract] OR "youths"[Title/Abstract] OR "teen"[Title/Abstract] OR "teens"[Title/Abstract] OR "teenage*"[Title/Abstract]) AND (NOTNLM OR publisher[sb] OR inprocess[sb] OR pubmednotmedline[sb] OR indatareview[sb] OR pubstatusaheadofprint)) NOT (("Animal" OR "animals" OR "rat" OR "rats" OR "mouse" OR "mice" OR "rodent*" OR "swine" OR "porcine" OR "murine" OR "sheep" OR "lamb" OR "lambs" OR "pig" OR "pigs" OR "piglet" OR "piglets" OR "rabbit" OR "rabbits" OR "cat" OR "cats" OR "dog" OR "dogs" OR "cattle" OR "bovine" OR "monkey" OR "monkeys" OR "trout" OR "marmoset" OR "marmosets") NOT ("human" OR "humans" OR "patient" OR "patients" OR "newborn*" OR "baby" OR "babies" OR "neonat*" OR "infan*" OR "toddler*" OR "pre-schooler*" OR "preschooler*" OR "kindergarten" OR "boy" OR "boys" OR "girl" OR "girls" OR "child" OR "children" OR "childhood" OR "adolescen*" OR "pediatric*" OR "paediatric*" OR "youth*" OR "teen" OR "teens" OR "teenage*" OR "school-aged*" OR "school-child*" OR "school-girl*" OR "school-boy*" OR "schoolgirl*" OR "schoolboy*" OR "man" OR "men" OR "woman" OR "women" OR "adult" OR "adults" OR "middle-age*" OR "elderly"))) AND (casereports[Filter])) OR (((("Posaconazole"[Title/Abstract] OR "noxafil"[Title/Abstract] OR "sch-56592"[Title/Abstract] OR "sch56592"[Title/Abstract] OR "spriafil"[Title/Abstract]) AND ("newborn*"[Title/Abstract] OR "new-born*"[Title/Abstract] OR "baby"[Title/Abstract] OR "babies"[Title/Abstract] OR "neonat*"[Title/Abstract] OR "neo-nat*"[Title/Abstract] OR "infan*"[Title/Abstract] OR "toddler*"[Title/Abstract] OR "pre-schooler*"[Title/Abstract] OR "preschooler*"[Title/Abstract] OR "kinder"[Title/Abstract] OR "kinders"[Title/Abstract] OR "kindergarten*"[Title/Abstract] OR "kinder-aged"[Title/Abstract] OR "boy"[Title/Abstract] OR "boys"[Title/Abstract] OR "girl"[Title/Abstract] OR "girls"[Title/Abstract] OR "child"[Title/Abstract] OR "children"[Title/Abstract] OR "childhood"[Title/Abstract] OR "pediatric*"[Title/Abstract] OR "paediatric*"[Title/Abstract] OR "school-age*"[Title/Abstract] OR "schoolage*"[Title/Abstract] OR "schoolchild*"[Title/Abstract] OR "schoolgirl*"[Title/Abstract] OR "schoolboy*"[Title/Abstract] OR "adolescen*"[Title/Abstract] OR "youth"[Title/Abstract] OR "youths"[Title/Abstract] OR "teen"[Title/Abstract] OR "teens"[Title/Abstract] OR "teenage*"[Title/Abstract]) AND (NOTNLM OR publisher[sb] OR inprocess[sb] OR pubmednotmedline[sb] OR indatareview[sb] OR pubstatusaheadofprint)) NOT (("Animal" OR "animals" OR "rat" OR "rats" OR "mouse" OR "mice" OR "rodent*" OR "swine" OR "porcine" OR "murine" OR "sheep" OR "lamb" OR "lambs" OR "pig" OR "pigs" OR "piglet" OR "piglets" OR "rabbit" OR "rabbits" OR "cat" OR "cats" OR "dog" OR "dogs" OR "cattle" OR "bovine" OR "monkey" OR "monkeys" OR "trout" OR "marmoset" OR "marmosets") NOT ("human" OR "humans" OR "patient" OR "patients" OR "newborn*" OR "baby" OR "babies" OR "neonat*" OR "infan*" OR "toddler*" OR "pre-schooler*" OR "preschooler*" OR "kindergarten" OR "boy" OR "boys" OR "girl" OR "girls" OR "child" OR "children" OR "childhood" OR "adolescen*" OR "pediatric*" OR "paediatric*" OR "youth*" OR "teen" OR "teens" OR "teenage*" OR "school-aged*" OR "school-child*" OR "school-girl*" OR "school-boy*" OR "schoolgirl*" OR "schoolboy*" OR "man" OR "men" OR "woman" OR "women" OR "adult" OR "adults" OR "middle-age*" OR "elderly"))) AND (comment[Filter] OR editorial[Filter] OR letter[Filter]))))] |
| **Cochrane: 2005 - 2023** | |
| Set | Search statement |
| 1. | “Posaconazole” OR “noxafil” OR “sch-56592” OR “sch56592” OR “spriafil” |
| 2. | “newborn*” OR “new-born*” OR “baby” OR “babies” OR “neonat*” OR “neo-nat*” OR “infan*” OR “toddler*” OR “pre-schooler*” OR “preschooler*” OR “kinder” OR “kinders” OR “kindergarten*” OR “kinder-aged” OR “boy” OR “boys” OR “girl” OR “girls” OR “child” OR “children” OR “childhood” OR “pediatric*” OR “paediatric*” OR “school-age*” OR “schoolage*” OR “schoolchild*” OR “schoolgirl*” OR “schoolboy*” OR “adolescen*” OR “youth” OR “youths” OR “teen” OR “teens” OR “teenage*” |
| 3. | ("Animal" OR "animals" OR "rat" OR "rats" OR "mouse" OR "mice" OR "rodent*" OR "swine" OR "porcine" OR "murine" OR "sheep" OR "lamb" OR "lambs" OR "pig" OR "pigs" OR "piglet" OR "piglets" OR "rabbit" OR "rabbits" OR "cat" OR "cats" OR "dog" OR "dogs" OR "cattle" OR "bovine" OR "monkey" OR "monkeys" OR "trout" OR "marmoset" OR "marmosets") NOT ("human" OR "humans" OR "patient" OR "patients" OR "newborn*" OR "baby" OR "babies" OR "neonat*" OR "infan*" OR "toddler*" OR "pre-schooler*" OR "preschooler*" OR "kindergarten" OR "boy" OR "boys" OR "girl" OR "girls" OR "child" OR "children" OR "childhood" OR "adolescen*" OR "pediatric*" OR "paediatric*" OR "youth*" OR "teen" OR "teens" OR "teenage*" OR "school-aged*" OR "school-child*" OR "school-girl*" OR "school-boy*" OR "schoolgirl*" OR "schoolboy*" OR "man" OR "men" OR "woman" OR "women" OR "adult" OR "adults" OR "middle-age*" OR "elderly") |
| 4. | (#1 AND #2) NOT #3 |

## Table S2: Bias assessment using the NOS tool

| Source | Selection | | | | Comparability | Outcome | | | Overall score^b^ |
| --- | --- | --- | --- | --- | --- | --- | --- | --- | --- |
|  | Representative  -ness of the cohort | Selection of the non-exposed cohort^a^ | Ascertainment of exposure | Demonstration that outcome of interest was not present at start of study | Comparability of cohorts on the basis of the design or analysis | Assessment of outcome | Was follow-up long enough for outcomes to occur | Adequacy of follow up of cohorts |  |
| Bernardo et al. 2020^22^ | * | N/A | * | * | N/A | * | * | * | 6 |
| Berardo et at. 2013^37^ | * | N/A | * | * | N/A | * | 0 | 0 | 4 |
| Döring et al. 2017^24^ | * | N/A | * | * | N/A | * | * | * | 6 |
| Döring et al. 2017^21^ | * | N/A | * | * | N/A | * | * | * | 6 |
| Döring et al. 2012^36^ | * | N/A | * | * | N/A | * | * | * | 6 |
| Duehlmeyer et al. 2021^25^ | * | * | * | * | * | * | 0 | 0 | 6 |
| Garner et al. 2021^26^ | * | N/A | * | * | N/A | * | 0 | 0 | 4 |
| Heinz et al. 2016^27^ | * | N/A | * | * | N/A | * | * | * | 6 |
| Jia et al. 2022^23^ | * | N/A | * | * | N/A | * | * | * | 6 |
| Krishna et al. 2007^2^ | * | N/A | * | * | N/A | * | * | * | 6 |
| Lai et al. 2020^28^ | * | N/A | * | * | N/A | * | * | * | 6 |
| Mathew et al. 2017^29^ | * | N/A | * | * | N/A | * | 0 | 0 | 4 |
| Mauro et al. 2020^38^ | * | N/A | * | * | N/A | * | * | * | 6 |
| McMahon et al. 2017^30^ | * | N/A | * | * | N/A | * | 0 | 0 | 4 |
| Takpradit et al. 2021^31^ | * | * | * | * | 0 | * | * | * | 7 |
| Tragiannidis et al. 2019^32^ | * | N/A | * | * | N/A | * | * | * | 6 |
| Vanstraelen et al, 2016^33^ | * | N/A | * | * | N/A | * | * | * | 6 |
| Vicenzi et al. 2018^34^ | * | N/A | * | * | N/A | * | * | * | 6 |
| Wass et al. 2020^35^ | * | N/A | * | * | N/A | * | 0 | 0 | 4 |

1. Assessed for studies comparing posaconazole to another antifungal.
2. The maximum overall score is 9 stars for studies where comparability was assessed and 7 stars for studies where comparability was not assessed (a maximum of 1 star can be earned for each item in the scale with the exception of comparability where a maximum of 2 stars can be earned). Studies with a score of 7 stars were considered to have a low risk of bias, and those with scores of ≤7 had a medium or high risk of bias. N/A = not applicable
